# Hemophagocytic Lymphohistiocytosis in a Patient with Classical Hodgkin Lymphoma

**DOI:** 10.1155/2016/2103612

**Published:** 2016-10-10

**Authors:** G. Hyun, K. J. Robbins, N. Wilgus, L. Grosso, S. D. Goyal

**Affiliations:** Saint Louis University School of Medicine, 1402 S. Grand Blvd, St. Louis, MO 63104, USA

## Abstract

*Introduction*. Hemophagocytic lymphohistiocytosis (HLH) is a rare hyperinflammatory syndrome that can be associated with inherited genetic mutations, malignancy, autoimmune disorders, and viral infections. Though the pathogenesis is not fully known, HLH is understood to be a reactive process in the setting of uncontrolled activation of macrophages, CD8+ cytotoxic lymphocytes, and other immune cells. Hallmark clinicopathological features of HLH include fevers, cytopenias, hepatosplenomegaly, and hemophagocytosis in the bone marrow.* Case Presentation*. A previously healthy 28-year-old Caucasian male presented with a one-month history of persistent fever, night sweats, and unintentional weight loss. He was diagnosed with classical Hodgkin Lymphoma (HL) by core-needle biopsy of an axillary lymph node. Both bone marrow involvement by HL and hemophagocytosis were seen on subsequent bone marrow biopsy. Other findings included pancytopenia, splenomegaly, and elevated serum ferritin. Extensive work-up for autoimmune and infectious etiologies was unremarkable. The patient had a complete response after chemotherapy with Adriamycin, bleomycin, vincristine, and dacarbazine.* Conclusion*. This report documents the exceedingly uncommon association between HLH and HL. HLH is a hyperinflammatory syndrome with high mortality, so it is imperative to identify and treat the underlying cause for secondary HLH. Malignancy-associated HLH should be considered in the differential diagnosis for cancer patients who present with fever, cytopenias, and splenomegaly.

## 1. Introduction

Hemophagocytic lymphohistiocytosis (HLH) is a hyperinflammatory syndrome mediated by the uncontrolled activation of immune cells (macrophages, lymphocytes, and histiocytes) and elevated cytokines such as tumor necrosis factor *α* (TNF-*α*), interleukin 6 (IL-6), interferon *γ* (IFN-*γ*), and macrophage inflammatory protein 1*α* (MIP-1*α*) [1]. Clinicopathological features include fever, cytopenias, splenomegaly, jaundice, neurological symptoms, and hemophagocytosis in bone marrow, liver, or lymph nodes [2].

The familial form of HLH is an autosomal recessive disease that typically presents during childhood and is diagnosed by identification of mutations in HLH-associated genes (*PRF1*,* UNC13D*,* STX11*,* STXBP2*,* Rab27A*,* SH2D1A*, or* BIRC4*) [3]. These mutations affect the exocytosis of cytotoxic granules in natural killer (NK) cells, leading to a hyperinflammatory state. Acquired HLH, however, can present at any age and is often secondary to malignancy, autoimmune disorders, or viral infections. Malignancy-associated HLH (M-HLH) can occur before, during, or after diagnosis of the primary malignancy [4].

Based on current guidelines published by the HLH Study Group of the Histiocyte Society in 2004 [5], five of the following eight criteria must be met for diagnosis: (1) fever, (2) splenomegaly, (3) peripheral blood cytopenia, with at least two of the following: hemoglobin < 9 g/dL; platelets < 100,000/*μ*L; absolute neutrophil count < 1000/*μ*L, (4) hypertriglyceridemia (fasting triglycerides > 265 mg/dL) and/or hypofibrinogenemia (fibrinogen < 150 mg/dL), (5) hemophagocytosis in bone marrow, spleen, lymph node, or liver, (6) low or absent NK cell activity, (7) ferritin > 500 ng/mL, and (8) elevated soluble CD25 > 2400 U/mL.

## 2. Case Presentation

A previously healthy 28-year-old Caucasian male presented to a local community hospital for one-month history of persistent fevers (*T*_max_ 39.4°C), night sweats, chills, and unintentional weight loss of approximately 15% from his baseline weight of 87 kg. The patient had no preexisting chronic medical problems. He had a 1.5 pack-year smoking history and stopped smoking five years ago. He did not drink alcohol or abuse illicit drugs. He lived with his parents and worked as a waiter. There was no family history of hematologic malignancy. He denied any recent history of travel.

**Figure 1:**
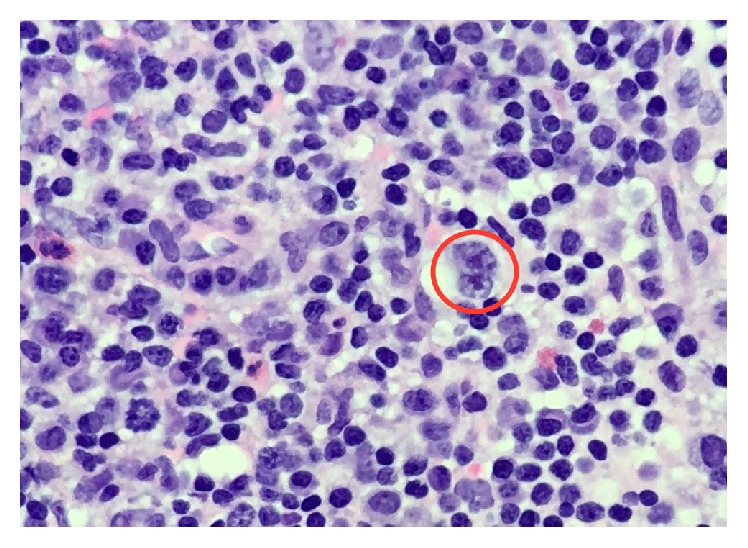
Core-needle biopsy of a right axillary lymph node revealed Reed-Sternberg cells (red circle) in a background of fibrosis and mixed inflammatory infiltrate.

After 2 recent brief hospitalizations in the prior month which were diagnostically unrevealing, he represented with persistent constitutional symptoms. Labs were remarkable for marked pancytopenia with a white blood cell count of 1,300/*μ*L, an absolute neutrophil count of 900/*μ*L, a hemoglobin concentration of 7.5 g/dL, and a platelet count of 15,000/*μ*L. Chemistry panel and liver function tests were unremarkable. Viral work-up was negative for influenza A/B, RSV, CMV, HIV, parvovirus B19, and hepatitis B virus. EBV was detected, but below the threshold for reliable quantification. Blood and urine cultures showed no growth. CT imaging showed diffuse adenopathy. Core-needle biopsy of a right axillary lymph node revealed Reed-Sternberg cells in a background of fibrosis and mixed inflammatory infiltrate (Figure 1).

**Figure 2:**
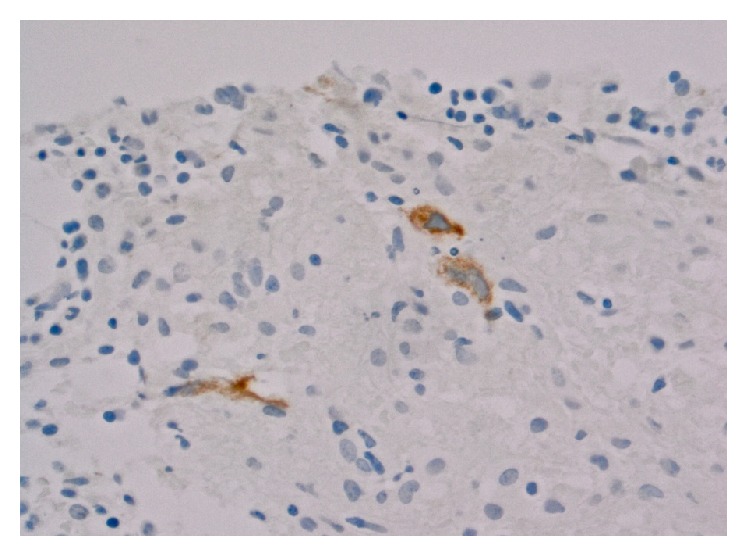
Bone marrow core showed foci of fibrosis with CD30-positive cells.

**Figure 3:**
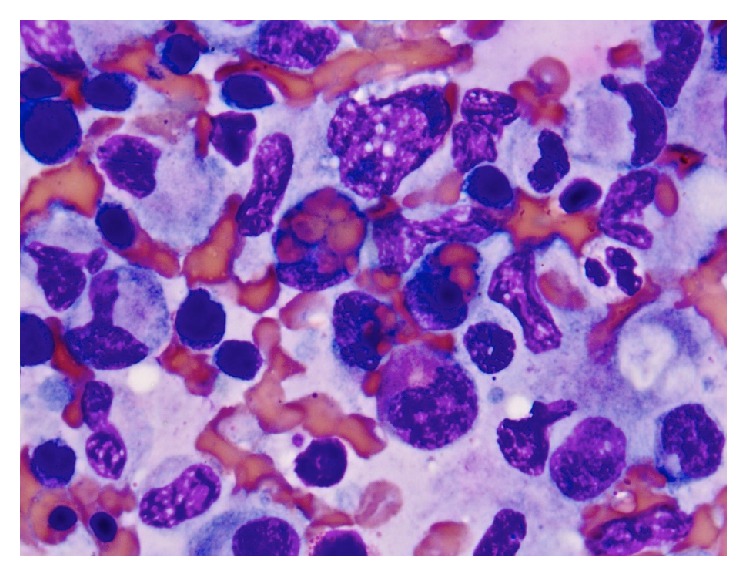
Hemophagocytosis consistent with HLH was noted in the bone aspirate smears.

The tumor cells showed expression of PAX-5 (weak), CD30, and CD15. Tumor cells were negative for CD20 and CD45. Epstein-Barr virus was expressed by the tumor cells. These features were diagnostic of classical HL. Bone marrow core biopsy revealed foci of fibrosis with rare CD30-positive cells, consistent HL (Figure 2). Additionally, hemophagocytosis was noted in the bone marrow aspirate smears (Figure 3). Further subclassification was not possible due to limited sample; excisional lymph node biopsy was not performed because it would have delayed treatment initiation.

**Figure 4:**
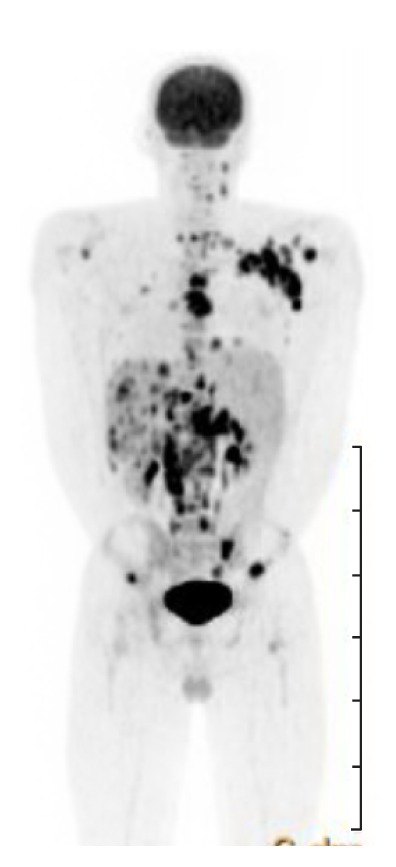
Pretreatment maximum intensity projection (MIP) scan of the whole body showed increased extensive lymphadenopathy above and below the diaphragm with multiple osseous foci of increased FDG uptake.

PET scan of the whole body showed extensive lymphadenopathy above and below the diaphragm with multiple osseous foci of increased FDG uptake, compatible with the patient's known diagnosis of HL (Figure 4).

The spleen was also noted to be enlarged at 17 cm craniocaudally but did not show increased FDG uptake on the pretreatment MIP scan. Additional laboratory work-up included an elevated ferritin at 1731 ng/mL, elevated fibrinogen at 449 mg/dL, normal triglyceride level at 124 mg/dL, and an elevated soluble IL-2 receptor of 1788 pg/mL.

Induction chemotherapy with Adriamycin, bleomycin, vincristine, and dacarbazine (ABVD) was initiated. The following day, the patient became hypothermic with a temperature of 34.4°C, but he improved after several hours of warm fluids and a warming blanket. Fevers never returned after the start of ABVD. By Cycle 1/Day 15 of ABVD treatment, counts had already markedly improved and continued to improve (Table 1).

**Figure 5:**
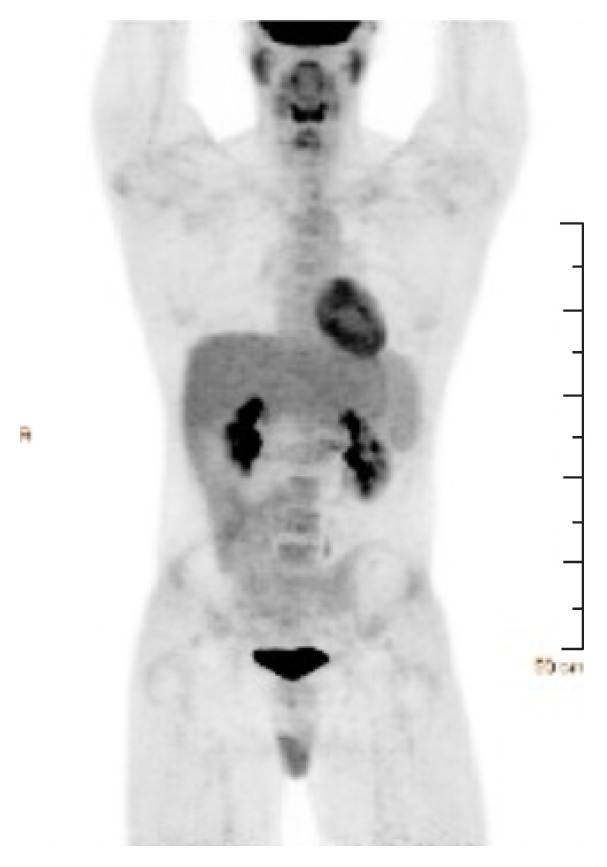
PET scan at Cycle 3/Day 1 showed interval resolution of FDG avid lymphadenopathy and bone lesions.

Interim PET scan on Cycle 3/Day 1 showed interval resolution of FDG avid lymphadenopathy in the neck, chest, and abdomen with resolution of bone lesions (Figure 5).

**Figure 6:**
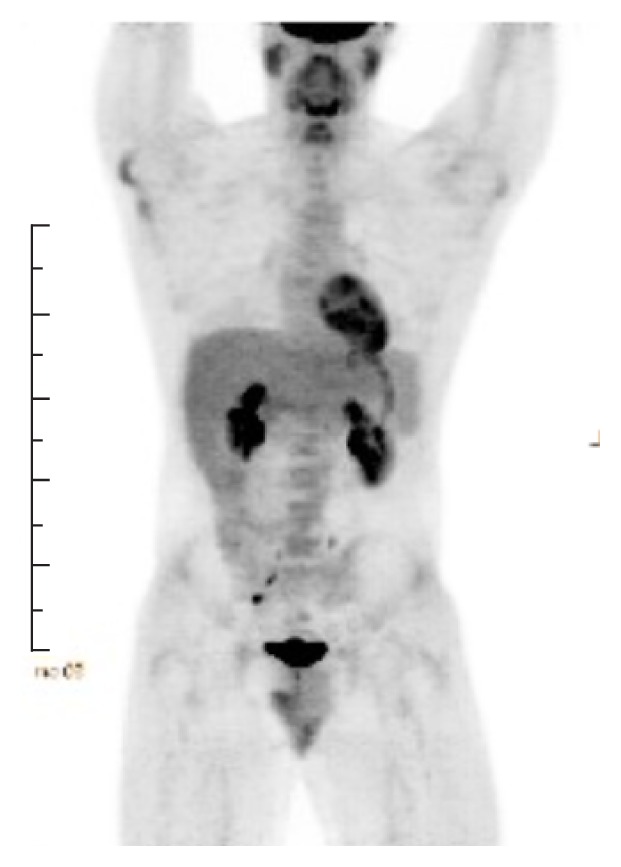
Restaging MIP scan showed complete response to treatment.

After six cycles of ABVD, restaging PET scan showed complete response to treatment with no residual or recurrent disease (Figure 6).

**Figure 7:**
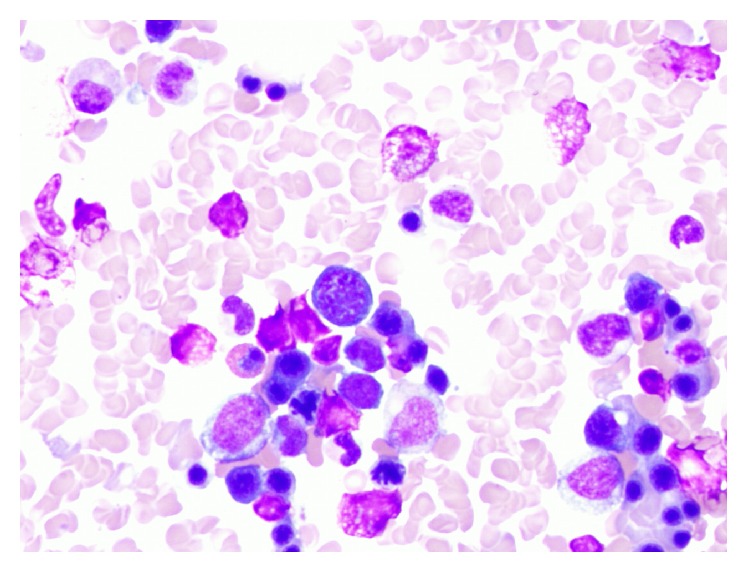
Posttreatment bone marrow biopsy showed mild hypocellularity with erythroid hyperplasia and dyspoiesis in the absence of Reed-Sternberg cells or hemophagocytosis.

Bone marrow biopsy revealed mild hypocellularity with erythroid hyperplasia and dyspoiesis (Figure 7).

There was no increase in blasts and no evidence of lymphoma, Reed-Sternberg cells, or hemophagocytosis. Flow cytometry was negative. The patient continued to improve clinically with improved counts (Table 2), increased appetite, and weight gain.

## 3. Discussion

To date, there is scarce literature published on M-HLH. M-HLH has been most commonly associated with hematological malignancies, particularly non-Hodgkin Lymphoma [6].

In a multicenter retrospective case series of 68 patients with HLH [7], Schram et al. found that the most common underlying disorder was malignancy (33/68, 49%) followed by infection (22/68, 33%), autoimmune disease (19/68, 28%), and idiopathic HLH (15/68, 22%). Of the M-HLH cases, 13 patients had B-lymphoid malignancies, 9 had myeloid malignancies, 9 had T-lymphoid malignancies, and 2 had solid tumors. Hodgkin Lymphoma was the most common B-lymphoid subtype (4/13, 31%). Of the infection-associated cases, viral infections were the most common etiology, most notably EBV and CMV. The observation that some patients had multiple underlying triggers suggests that an acute infection in the setting of an immune system already impaired by malignancy or autoimmune disease may play a role in the pathogenesis of HLH.

Consistent with other studies [8, 9], patients with M-HLH had a worse prognosis than those without cancer (median survival 2.8 months versus 10.7 months, *p* = 0.007). Schram et al. found that there was no statistical difference in overall survival in patients with M-HLH who were treated with etoposide. However, this may have been due to limited sample size and bias in giving etoposide to patients who were failing standard chemotherapy.

In a retrospective study conducted in Sweden, Machaczka et al. found that eight out of 887 patients with hematological malignancies developed M-HLH, an incidence of approximately 1/280,000 per year. Three patients presented with HLH as first manifestation of unknown malignancy while 5 developed HLH during therapy for known malignancy. Two patients received immunosuppressive therapy (IVIG, corticosteroids) and died without improvement. Of the six who were treated with a modified HLH-94 protocol (etoposide, corticosteroids), three achieved remission while the rest did not respond to treatment and died within an average of 2.4 months after M-HLH diagnosis. The most common and lethal complication in these immunocompromised patients was infection, which exacerbated the severity of HLH by further activation of an already prolonged and excessive inflammatory state [4].

## 4. Conclusions

HLH should be considered in the differential diagnosis for adult patients with malignancy who present with recurrent fevers, splenomegaly, and pancytopenia. Our patient was diagnosed according to the HLH-2004 guidelines with Stage IVB classical HL with concurrent HLH. International Prognostic Score (IPS) was 5, which is associated with a 5-year progression-free survival (PFS) of 42% and overall survival (OS) of 56%. He achieved a complete response after six cycles of ABVD chemotherapy. He also had a rapid and complete resolution of clinical symptoms and cytopenias which were presumably due to HLH. Given the rarity of HL associated with HLH, we cannot speculate on whether the HLH would adversely impact the long-term outcomes projected by the IPS score. Further work is also needed to understand the pathogenesis of HL-associated HLH and to determine the role of environmental triggers in the development of secondary HLH.

## Figures and Tables

**Table 1:** Patient's improving lab values following two cycles of ABVD chemotherapy.

	Ref. range	Cycle 1 Day 1	Cycle 1 Day 15	Cycle 2 Day 1
WBC	Latest range: 3.5–10.5 10^3^/*μ*L	1.0 (L)	1.6 (L)	2.0 (L)
Hemoglobin	Latest range: 13.5–17.5 g/dL	7.1 (L)	10.6 (L)	11.4 (L)
Hematocrit	Latest range: 39.0–50.0%	20.7 (L)	31.9 (L)	33.3 (L)
Platelets	Latest range: 150–400 10^3^/*μ*L	15 (LL)	181	196
Neutrophil ABS	Latest range: 1.6–7.0 10^3^/*μ*L	0.7 (L)	0.26 (LL)	0.9 (L)

**Table 2:** Patient's lab values following six cycles of ABVD chemotherapy.

	Cycle 3 Day 1	Cycle 4 Day 1	Cycle 5 Day 1	Cycle 6 Day 1	After 6 cycles Follow-up
WBC	2.7 (L)	1.7 (L)	1.7 (L)	1.6 (L)	4.8
Hemoglobin	12.4 (L)	11.9 (L)	12.6	12.9	13.6
Hematocrit	37.5 (L)	35.3 (L)	36.6	37.7	40.5
Platelets	237	165	179	194	166
Neutrophil ABS	1.0 (L)	0.49 (LL)	0.56 (L)	0.25 (LL)	3.04

## Abbreviations

HLH: Hemophagocytic lymphohistiocytosis
M-HLH: Malignancy-associated hemophagocytic lymphohistiocytosis
HL: Hodgkin Lymphoma.
